# Supplementary material for: Are carbon emissions trading and green financial instruments synergistic? -Comprehensive quantitative research based on content analysis
Source: PLoS One. 2024 Mar 7;19(3):e0298601. doi: 10.1371/journal.pone.0298601 (PMC10919601; doi:10.1371/journal.pone.0298601)
Supplement: S1 Data — (ZIP) [file pone.0298601.s001.zip › Data/地级市Z_F工作报告.zip!/║╙─╧/╨┬╧τ╩╨/└┤╫╘╣1⁄2╓┌║┼í╛┬φ┐╦╩2╛▌═°í┐_2006.docx]

各位代表：

　　现在，我代表市人民政府向大会做工作报告，请予审议，并请各位政协委员和列席人员提出意见。

一、2005年及“十五”以来的工作

　　2005年，市人民政府在省委、省政府和市委领导下，团结带领全市人民，坚持科学发展观，锐意进取，扎实工作，较好完成了市十届人大二次会议确定的各项目标任务。

　　经济发展的速度与质量同步提高。按照新口径核算，全市完成生产总值543亿元，增长14.1%。一、二、三次产业分别增长8.8%、17.6%、11.9%。全市粮食总产量310万吨，增长4.9%；肉、蛋、奶产量分别增长8%、8.8%、46.5%。限额以上工业实现增加值175亿元、利润23.2亿元，分别增长24.2%、61.8%。工业综合效益指数142，提高21.3 。地方财政一般预算收入25.4亿元，增长32.8%。金融机构各项存款余额560.7亿元，增长13.7%；贷款余额322.2亿元，增长9.5%。

　　经济结构调整步伐加快。三次产业比例调整为16.4:51.6:32，二次产业比重提高2.6个百分点。非公有制经济比重达到55%，提高4.7个百分点。城镇化率达到33.7%，提高1.7个百分点。农业结构进一步优化。优质农产品比重提高，绿色食品生产基地面积和通过绿色食品认证的农产品数量列全省第一，无公害畜产品生产基地数量居全省第一，畜牧业产值占农业总产值比重44.3%，提高4.1个百分点。工业经济集中度和科技含量明显提高。50户重点企业产值占全市工业总产值比重达50%，高技术产业增加值占工业增加值比重达10.5%。现代服务业发展迅速。光彩大市场等物流项目开工建设。万仙山、京华园景区通过4A级景区验收，关山景区被认定为国家地质公园，八里沟进入全省接待人数前十名景区。社区服务网点达到954个。邮电通信、金融保险业分别增长12.3%、11%。

　　固定资产投资快速增长。全社会固定资产投资319.6亿元，增长46.5%，其中工业投资147亿元，增长55%。实施千万元以上项目647个，增加267个，当年完成投资225亿元。全市138个重点项目中，新飞集团年产4000辆专用汽车、白鹭化纤年产1.5万吨差别化新型连续纺粘胶长丝、平原和天瑞公司日产5000吨干法水泥等30个项目建成投产。宝山电厂2×66万千瓦火电、金龙集团年加工6万吨精密铜管等45个项目顺利开工。

　　城乡基础设施更加完善。完成市区到卫辉快速路建设，新建改造干线公路116公里，改造县乡道路1500公里，在建高速公路179公里。新扩建牧野路、兴隆路等21条26.6公里城市道路，整治小街巷108条，市区40.5公里环路全部贯通，新区20平方公里路网初具雏形。市垃圾处理场投入运行，小尚庄污水处理厂基本建成，县（市）污水处理厂均已开工。新建改造供水管网57.2公里。城市气化率达到80%。城区热网开工建设。市区绿化覆盖率达到35.7%，人均绿地面积达到6.5平方米。新建改造公厕62座。市人民公园免费开放。商品房竣工107.2万平方米，增长62.6%。6个县实现省级平原绿化高级达标。农田水利基本建设继1998年之后重获省“红旗渠精神杯”。

　　改革开放取得新成绩。列入省考核范围的国有企业完成和基本完成改制121户，占总数的77.6%。产权结构多元化的粮食购销体制初步形成。农村信用社改革成效显著。完成乡镇机构改革，撤并乡镇29个，清退临时人员2397名，分流超编人员7913名。交通系统“三项改革” 基本完成，建设系统企事业单位改革有序推进。对外开放跃升到新的层次。成功举办深圳海外客商春节团拜会暨新乡市情说明会，组织参加豫港投资贸易洽谈会等经贸招商活动，签约项目82个，总投资108.3亿元。美国沃尔玛、英国联合生命等4户世界500强企业和香港森仕制衣、广东立白集团、深圳海滨制药等一批国内外知名企业落户新乡。新批外商投资企业35家，合同利用外资8631万美元，增长30%；实际利用外商直接投资6016万美元，增长32%；利用市外资金48亿元，增长59%。进出口总额5.3亿美元，增长36.2%；其中出口3.5亿美元，增长32.3%。

　　人民生活水平继续提高。社会消费品零售总额164亿元，增长14.3%。城镇居民人均可支配收入8312元，增长16.3%；农民人均纯收入3133元，增长14%。全市筹措用于改善民生方面的资金56.9亿元，增长16%。新增就业17.5万人，安置下岗失业人员7.1万人，其中“4050”人员1.9万人；发放下岗职工小额贷款1亿元。养老保险金和失业保险金均按时足额发放。实现失业保险市级统筹。进一步规范城市低保制度，全面推行农村低保。333.6万农民参加新型农村合作医疗，参合率82%。全省第一家实施全区域城市医疗救助试点。全市农民通过“一免三补”政策减负增收3.6亿元。完成664间倒房重建任务，受灾群众入冬前全部搬进新居。新增农村户用沼气池8.6万个。超额完成建设领域拖欠工程款和农民工工资清欠任务。

　　社会事业协调发展。新增高新技术企业33 家、省级企业研发中心1个、企业博士后科研工作站1个。专利申请突破1000件，居全省第2位。科技进步贡献率达到42%。发放农村基础教育 “两免一补”资金5076万元，资助农村学生42万人次。投资7000余万元开工建设农村中小学现代远程教育工程。完成845所农村中小学危房改造，新建校舍41万平方米。河南师大和市委党校新校区开工建设。大力推进文化体制改革，组建电广传媒有限责任公司，金环文化发展有限公司被命名为省首批12家文化产业示范基地之一，“四庙一馆”保护开发工程进展顺利。　　市医疗废物集中处置中心开工建设。水、大气污染治理取得新成效。煤炭资源整合工作全面完成。空心村、砖瓦窑及工矿废弃地“三项整治”置换土地指标11500亩。实施计划生育家庭奖励扶助政策，人口自然增长率控制在5.1‰以内。审计工作在全省名列前茅。史志工作进入全国先进行列。新闻出版、防震减灾、统计、外事、侨务、档案、气象、测绘、人防等工作都取得新的成绩。

　　精神文明和民主法制建设得到加强。认真办理人大代表议案、建议和政协委员提案。顺利完成第五届村委会换届选举。扎实开展保持共产党员先进性教育活动。认真落实党风廉政建设责任制，廉政建设和反腐败斗争取得新成效。广泛开展“道德规范进万家”活动，推进未成年人思想道德建设。荣获省级文明城市工作先进市。民族团结进步教育活动取得较好成效。健全信访党政联席会议、领导包案、市领导接访等制度，认真解决群众反映突出的问题。强化安全生产责任制，深入开展专项整治，安全生产形势平稳。加强社会治安综合治理，“严打”斗争扎实有效，治安防控能力进一步提高，社会大局保持稳定。

　　在市十届人大二次会议上向全市人民承诺办好的10件实事，经过一年努力，基本得到落实。免征农业税、对种粮农民直接补贴等政策全部落实到位；实施“引石济卫”，卫河、人民胜利渠水质明显改善；卫河公园改造、科技文化公园及牧野广场二期绿化工程基本完成；人民医院综合病房楼、传染病应急医疗救治病房楼、新区公疗医院及14所中心乡镇卫生院、99所一般乡镇卫生院改扩建项目基本完工，市疾病预防控制中心、中心医院外科病房楼主体竣工；全市10.9万名增资退休人员按新标准领取养老金；完成经济适用房9.4万平方米，新增享受廉租房补助家庭100户；各县（市、区）教师工资和乡镇基层干部工资均按标准发放；老年活动中心、老年大学综合楼正在加紧建设；解决了重污染区114个行政村16.3万人的饮水安全和山丘区1.5万人的饮水困难问题，完成山区移民扶贫720人；关闭造纸企业制浆能力44.3万吨和86条水泥立窑生产线，取缔25家“十五小”企业。省委、省政府部署的10件实事和市委、市政府在保持共产党员先进性教育活动中承诺办好的10件实事、要解决好的10个突出问题也都得到较好落实。

　　2005年各项目标任务的顺利完成，标志着“十五”期间我市经济社会发展取得了具有重要意义的突破和跨越。预计全市生产总值年均增长12.2%，高出全省平均水平0.8个百分点；与“九五”末相比，地方财政一般预算收入和财政总支出均翻了一番，经济总量增长91%；累计完成固定资产投资总额是“九五”时期的3倍，综合经济实力显著增强。经过五年的努力，我市经济社会发展站在了新的战略起点上，进入全面提升产业层次、加快经济转型、促进统筹协调发展的新阶段。

　　回顾“十五”期间尤其是近年来的工作，有以下五点体会： 一是坚持以加快发展为主题，努力在区域竞争中争取主动。始终把发展作为第一要务，以“晋位升级”目标统一全市思想，主动顺应宏观调控，全力以赴抢抓发展机遇，众志成城冲破发展瓶颈，依靠技术创新，推进产业结构优化，克服自然灾害、原材料涨价等不利因素，保持经济社会的持续快速健康发展。 二是坚持以项目统领经济工作全局，增强经济社会持续发展能力。累计完成固定资产投资861亿元，实施千万元以上项目1082个，其中亿元以上项目126个。以五大强势产业和七大特色产业为主的工业体系初步形成，工业经济竞争力明显增强。农村基础设施更加完善，农业综合生产能力进一步提高。传统服务业得到提升，现代服务业发展加快。拉大城市框架，以交通道路为主的城乡基础设施建设得到加强，生态和人居环境明显改善。 三是坚持改革开放，增强经济发展活力。坚持社会主义市场经济的改革方向，不断把改革向纵深推进、向广度拓展，一些重点领域和关键环节的改革取得突破，体制机制环境进一步优化。按照“开明、开放、开发”的要求，着眼国际国内两个市场，举全市之力招商引资，打造承接东部产业转移的“桥头堡”。五年新批外商投资企业116家，利用外商直接投资2.3亿美元，外贸进出口总额14.9亿美元，对外开放意识和经济外向度明显提高。 四是坚持以人为本，促进社会和谐稳定。始终把帮扶弱势群体等作为政府的基本施政理念，累计投入惠民资金102.3亿元，占财政总支出一半以上。争取中央、省资金投入比“九五”期间增长3.6倍，市本级资金投入增长6.2倍。在全国率先全面实施新型农村合作医疗制度，全省首家实现养老保险和失业保险市级统筹，提前一年完成农村中小学库内D级危房改造任务。累计新增就业岗位25万个，帮助12.2万人实现脱贫，城乡人民生活水平不断提高。社区建设走在全国前列，各项社会事业全面发展。深入开展安全城市创建活动，社会大局保持稳定。 五是坚持推进责任、法治、服务型政府建设，不断优化发展环境。行政审批事项由1699项减少到260项，市、县两级行政服务中心规范运行。扩大政务公开，严格依法行政，组建经济发展环境110，实行行政首长问责制，加强行政效能监察，建立健全责任、有序、高效的工作机制，行政效率和服务水平进一步提高。扎实推进“五城”联创，成功创建“中国优秀旅游城市”，被中宣部定为全国信用建设试点市。坚持以事业凝聚人心，自觉维护班子团结、干群团结，形成了人和政通、人心思进的良好局面，营造了同心同德干事业、一心一意谋发展的浓厚氛围。

　　各位代表，2005年和“十五”时期所取得的成绩来之不易，主要归功于省委、省政府和市委的正确领导，归功于市人大、市政协的监督和支持，归功于全市人民的辛勤劳动。在此，我代表市人民政府，向各位人大代表和政协委员，向广大工人、农民、知识分子、干部、驻新解放军指战员、武警官兵、公安干警及社会各界人士致以崇高的敬意！向关心、支持新乡建设的香港特别行政区同胞、澳门特别行政区同胞、台湾同胞、海外侨胞和国际友人表示衷心的感谢！

　　在肯定成绩的同时，我们也清醒地看到我市经济社会发展中存在的一些突出矛盾和问题： 一是经济结构性矛盾没有得到根本解决，整体竞争力不强。我市加工工业占全部工业比重近90%，在能源和原材料价格居高不下、产成品价格难以联动上升的情况下，企业盈利能力大幅下降。 二是国企改革进入攻坚阶段，改革成本过高，推进困难。 三是农村经济基础依然比较薄弱，农民增收的长效机制尚未建立，农民持续增收困难。 四 是财政收支矛盾突出，公益性、基础性支出与人民群众不断增长的需求还有较大差距。 五是就业和社会保障任务仍很艰巨，困难企业职工安置等方面压力很大。 六是关系群众切身利益的上学、就医等问题仍然突出。 七是信访工作任务较重，社会矛盾排查调处机制仍需完善，社会治安防控体系还需健全。 八是一些干部工作标准低、作风不扎实，官僚主义、形式主义、庸俗作风等还不同程度存在。对以上问题，我们将在今后工作中着力加以解决。

二、“十一五”发展的总体思路和奋斗目标

　　“十一五”时期是加速推进工业化、城镇化和农业现代化，为在全省率先实现全面建设小康社会目标奠定基础的关键时期。我市经济社会发展要以邓小平理论和“三个代表”重要思想为指导，坚持以科学发展观统领经济社会发展全局，紧紧围绕全面建设小康社会的奋斗目标，坚持以人为本，坚持把加快发展作为第一要务，坚持把不断提高人民生活水平作为根本出发点，加快转变经济增长方式，积极推进经济结构调整，大力发展循环经济，建设资源节约型和环境友好型社会，着力构建和谐社会。以增强综合经济实力、自主创新能力、经济可持续发展能力、区域经济竞争力为主线；强力推进工业化、城镇化、农业现代化、民营经济规模化、服务业现代化、国民经济和社会信息化进程；贯彻落实项目带动、开放驱动、改革推动、科教兴市、优化环境、人才强市、健全保障和可持续发展举措；打造中原地区加工工业基地、高素质人力资源培育基地和区域现代物流中心、现代农业示范中心。把新乡建设成为经济发达、政治民主、社会和谐、文化先进、人民富裕、环境优美、文明进步的中原城市群强市。

　　“十一五”经济社会发展的主要奋斗目标是：

　　――经济持续快速健康发展，综合实力再上新台阶。全市生产总值年均增长12%以上，力争达到1000亿元，在全省的位次有所前移；财政一般预算收入年均增长13%以上，力争翻一番；三次产业结构优化调整为10.7:59.1:30.2；城镇化率达到43%。

　　――各项改革顺利推进，开放型经济建设取得新突破。坚持市场化取向改革，社会主义市场经济体制更加完善。完成国有集体企业改制任务，非公有制经济比重达到85%以上；不断扩大对外对内开放，累计实际利用外资6亿美元以上，利用市外境内资金350亿元；2010年进出口总额达到12.1亿美元，其中出口8亿美元。

　　――生态环境得到改善，资源节约型和环境友好型社会建设取得新进展。单位生产总值能耗比“十五”末降低20%左右，资源利用效率显著提高；森林覆盖率达到28%；城市生活污水处理率达到90%，垃圾无害化处理率达到100%，建成区绿化覆盖率达到40%，人均公共绿地面积达到11平方米。

　　――社会事业全面发展，和谐社会建设取得新成效。科技基础设施和技术创新能力进一步加强，科技进步贡献率达到48%。城镇基本普及高中阶段教育，职业教育在校生人数达到10万，高校在校生人数达到15万。文化强市建设取得突破性进展。公共卫生覆盖面扩大，新型农村合作医疗人口覆盖率达到95%。社会就业比较充分，社会保障体系比较健全，城乡居民生活质量和水平进一步提高。城镇居民人均可支配收入年均增长10%，农民人均纯收入年均增长7%；市区供热普及率达到70%；所有行政村通班车，解决农民 “出行难”问题。民主法制和精神文明建设进一步加强，公民思想道德素质、科学文化素质和健康素质明显提高。

三、2006年工作的主要目标和任务

　　2006年是“十一五”的开局之年，要以引资、项目双带动为主战略，增强经济发展后劲；以转变经济增长方式为主线，提高自主创新能力，发展循环经济，推进经济转型和结构调整；以中原城市群新乡都市区建设为载体，促进工业化、城镇化与社会主义新农村建设的良性互动；以国企改革攻坚和事业单位改革为重点，创造体制环境新优势；以关注民生和维护稳定为核心，着力构建和谐社会。主要目标是：生产总值增长13%；全社会固定资产投资增长20%；社会消费品零售总额增长14%；地方财政一般预算收入增长15%；全市城镇居民人均可支配收入增长9%，农民人均纯收入增长7%；单位生产总值能耗降低5%左右；城镇登记失业率控制在4.5%以内；人口自然增长率控制在6.5‰以内。

　　重点抓好六项工作：

　　（一）积极实施引资、项目双带动主战略，增强经济发展后劲

　　把引资、项目双带动战略放在经济工作首位。招商引资和项目建设是促进我市经济结构调整、实现经济全面提速的首要推动力。要继续坚持以项目统领经济工作全局，把招商引资作为项目建设投资的主渠道，进一步健全组织体系，完善工作机制，严格落实招商引资“五职”责任人制度和重点项目首席服务官制度，兑现奖惩，强力推进，务求实效。

　　进一步扩大引资和进出口规模。结合我市特点，围绕资源型、上下游产品衔接型、战略布局型、劳动密集型、市场占领型、能源材料消耗型六类企业的扩张需求，采取小分队招商、敲门招商、会展招商、以商招商等多种方式，有针对性地开展招商引资。建立战略投资者档案库，强化企业招商引资主体地位，鼓励支持市内企业拿出优质资产、优势项目吸引战略投资伙伴。制定项目寄养政策，建立项目用地、利税分配协调机制，保证项目有地可落，促进项目聚集。积极做好参加第四届（中国）河南投资贸易洽谈会、福州海峡两岸经贸洽谈会等招商活动的组织工作。力争新批外商投资企业30家，合同利用外资1亿美元，实际利用外资1亿美元，利用市外资金60亿元。积极组织参加广交会、东盟国际博览会等重点出口产品交易会，扩大高新技术产品和机电产品出口，进出口总额达到6.2亿美元。

　　切实加大项目实施力度。计划实施投资千万元以上项目556项，总投资874亿元，年度投资297亿元。其中重点项目140项，总投资533亿元；当年争取建成投产30项，完成投资134亿元。一是续建宝山电厂2×66万千瓦火电、环宇集团日产10万安时动力锂电池等90个总投资356亿元的重点项目，确保心连心化工年产30万吨尿素、华兰生物系列疫苗等30个项目建成投产。二是开工建设新飞集团年产万辆专用汽车工业园、赵固煤田二矿等50个总投资177亿元的重点项目。三是推进宝山电厂4×100万千瓦火电、宝泉抽水蓄能电站二期等重点项目的前期工作。

　　（二）加快农业现代化进程，积极建设社会主义新农村

　　加强社会主义新农村的规划与建设。依据“生产发展、生活宽裕、乡风文明、村容整洁、管理民主”的总体要求，按照城乡一体化目标，认真编制建设规划，优先支持县城及产业聚集区内的新农村建设。大力实施“六小工程”，完善农村基础设施。新建农村道路600公里，新增“村村通”行政村400个。继续实施大功、韩董庄、石头庄、沧河四大灌区节水续建配套工程。完善引黄工程体系，创新管理体制，提高引水效益。新发展沼气用户6万户。解决好9万人的饮水安全问题。优化农村电网，完善村民居住点供水、排水、垃圾集中堆放等生活设施。加快建立村级道路、街道环境管养保洁体制，积极探索通过 “一事一议”加强农村公益设施建设的新机制。

　　千方百计增加农民收入。调整种植业结构，抓好优质小麦基地及种子基地建设，扩大优质粮食和高效农作物比重。推进沿京广线优质猪禽生产带、黄河滩区绿色养殖带建设。新建速生丰产林基地10万亩、沿山经济林基地3万亩。围绕富民强县目标，壮大县域经济，发展二、三产业，促进农民就业创业。完善劳务输出体系，加强农民技能培训，实现劳务输出70万人次、劳务收入30亿元。坚持“多予、少取、放活”的方针，落实各项支农政策。加大扶贫开发力度，抓好97个村的整村推进和山区725人的扶贫搬迁。

　　大力发展农村公益事业。推进乡镇便民服务中心、农村社区服务中心等公共服务设施建设，完善农村低保、“五保户”供养、灾民救助等农村社会保障体系，推进新型农村合作医疗健康运行。健全重大动物疫病防控体系和农业技术推广体系。开展争创省文化先进县、乡（镇）活动，建设647个文化大院。实施广播电视村村通工程。广泛开展理想信念、科技文化和政策法纪三项教育，引导农民树立先进的思想观念和良好的道德风尚。完善村民自治机制，增强民主意识。

　　（三）努力提高自主创新能力，切实加快产业结构调整

　　建立健全自主创新体系。以服务支柱产业和先导行业为重点，以科技体制改革为动力，以科技条件和环境建设为基础，构建企业为主体、产学研结合的区域性创新体系，建设创新型城市。大力开展专利兴业、名牌兴市活动。重点推进电子信息、新材料、生物与新医药等方面的高新技术产品开发。新增市级和省级企业技术中心各3家、省级高新技术企业13家、省级高新技术产品14个。科技进步贡献率达到43%。

　　着力培育壮大优势产业。在支持电子电器、生物与新医药、环保电池及原材料、化纤纺织、能源电力五大强势产业和汽车及零部件、食品加工、机械装备、造纸、新型建材、医用卫材、化工七大特色产业加快发展的同时，重点推进纺织行业的资源整合与发展，力争销售收入增长25%；规模发展汽车及零部件产业，力争销售收入增长30%；继续壮大食品工业，力争销售收入增长35%；大力发展特色装备制造业，力争销售收入增长23%；发挥我市制冷产业规模、技术和市场优势，做好压缩机项目的引进和实施，拉长产业链条，形成产业聚集。

　　支持重点企业做大做强。完善激励机制，实行动态管理，加大服务力度，支持以11户省重点企业为核心的50户重点企业提速发展，着力培育大型企业集团。力争金龙集团年销售收入达到80亿元，新飞电器（集团）达到50亿元，白鹭化纤、新航集团和安美电子突破20亿元，环宇集团、刘庄农工商、新亚集团突破15亿元，五得利新乡分公司、心连心化工、科隆电器、卫华起重、孟电集团、豫新发电公司突破10亿元。充分发挥省级高新技术开发区、小店工业区、新乡经济技术开发区、国家级化学与物理电源产业园等园区的平台作用，加快金龙、新飞、白鹭等七个重点企业园区建设，促进产业集聚发展。积极推进标准厂房建设，争取建成20万平方米。

　　加强经济运行的综合协调与监控。做好煤、电、油、运的供求衔接，保证重点行业、重点企业、重点项目需求。继续举办银企合作洽谈会，积极争取金融部门信贷支持。对市直国有及国有控股、参股和集体工业企业逐户签订发展目标，定期督查，重点抓好盈利大户增盈和亏损大户扭亏工作。

　　促进服务业快速发展。加快发展现代服务业，引进新型管理模式和经营业态，着力发展第三方物流，积极推进小店和桥北综合物流园区、新华粮食物流中心等重点项目建设。大力发展文化产业，加快河南省文化产业示范基地建设，筹建文化图书产业物流交易中心。完善旅游基础设施，加大宣传推介和行业管理力度，创建4A级、3A级景区各1家。积极发展社区服务业、信息服务业和中介服务业。壮大提升商贸流通业，加快沃尔玛、东方商业文化步行街等商业设施建设。按照“健康、合理、方便群众”的原则，调整现有商业业态和区域布局结构。

　　（四）启动中原城市群新乡都市区建设，加速城镇化进程

　　 完善中原城市群新乡都市区规划。按照“一城三区五星环绕两个副中心”的规划要求，逐步调整都市区内卫星城的规模结构和空间布局，形成特色鲜明、组团有序、多层次、网络化的城镇体系。以重要交通干线为纽带，以城镇为载体，整合区域资源，加强分工协作，促进产业集聚，构建“一轴五带三级三十六个聚集区”，为招商引资搭建平台，加速工业化、城镇化和农业现代化进程。

　　启动都市区及拓展区基础设施建设。都市区及拓展区规划范围内的县城和各个聚集区内不低于3平方公里的起步区建设要全面启动，着力完善路网、电网、信息网、供水、排水、供气、污水处理、垃圾处理八项功能。加快推进黄河三桥及与107连接线至新晋高速道路、桥北新区起步区、新亚纸制品区起步区、一轴五带八条快速路绿化美化亮化等工程建设。

　　抓好城建交通六大工程。实施路网工程。续建济东高速、阿深高速新乡段，开工建设新晋高速新乡段和安新高速改扩建工程。新改建干线公路24.8公里、县乡公路200公里、城市道路9.6公里。实施园林绿化工程。建成新区公园等10处公共绿地、市区7条道路绿地及4个城市外围防护绿地项目，开工建设凤凰山省级森林公园。实施河渠工程。完成黄水河、镜高涝河截污导流及卫河市区段水系景观配套工程。实施环保工程。完成城市生活垃圾无害化处理场一期附属工程、市医疗废物集中处置中心和粪便无害化处理场，加快各县（市）污水处理和垃圾处理项目建设。实施大型公共设施工程。推进和平桥区域、汽车东站区域等“十大特色建筑群”建设，确保商行大厦、华电大厦等20个建筑工程开工。加快城市热网建设，力争卫河以北一半以上居民实现集中供暖。实施住宅工程。房地产新开工100万平方米以上，竣工50万平方米以上。

　　提高城市管理的层次和水平 。推行“单元网格”和“城市部件”先进管理办法，强化城管指挥和监督中心职能，促进管理规范化、制度化、长效化。严格执行城市规划，确保城市规划审批执行率100%。深化“五城”联创，推进国家卫生城市、国家园林城市创建工作。完善鼓励农民进城的优惠政策和城镇社保体系，实施“城中村”改造，吸引农村人口向城镇聚集，城镇化率提高1.7个百分点。

　　（五）继续深化各项改革，增强经济发展的动力和活力

　　集中力量实施国有企业改革攻坚。完成市属国有及国有控股、市属集体企业改制和企业学校移交任务。健全国有资产监管体系，完善国有企业法人治理结构，严格落实国有资产经营目标责任制，加强股权收益管理和产权代表管理，完善责任追究制度，制定行政事业和集体资产管理办法，确保国有资产保值增值。

　　推进财税金融体制改革。推行乡财县管与村财乡监。完善政府采购制度，严格国库集中支付，降低行政运行成本。深化部门预算改革，探索绩效预算制度，建立和完善编制管理与财政预算管理配套协调的约束机制。开展税收分析评估，实施纳税人分类管理，加大核定征收和税收稽查力度，严厉打击偷逃税。加快市商业银行向现代股份制商业银行转变。继续做好农村信用社清收不良贷款工作。推进投资体制改革，规范企业投资项目核准制和备案制，落实企业投资自主权。

　　深化其他各项改革。推进文化体制改革，加快国有文化企业公司制改造和经营性文化事业单位转企改制步伐。实行事业单位登记管理制度。深化市政公用行业改制工作。稳妥推进“村改居”和社区办事处体制改革。巩固乡镇机构改革成果，推进乡镇机构职能转变。完善征地制度，健全对被征地农民的合理补偿机制。全面完成国有粮食购销企业资产重组改制任务。

　　（六）始终坚持以人为本，努力构建社会主义和谐社会

　　建设资源节约型、环境友好型社会。认真做好全市新一轮土地利用总体规划修编工作，盘活存量建设用地，提高土地集约利用水平。引导火电、造纸、建材、纺织等行业发展循环经济，培育试点单位和示范企业，建设循环经济型工农业园区。整顿和规范矿产资源开发秩序，加快矿产资源整合。全面推行清洁生产，从严控制高耗能产业发展，坚决淘汰落后生产能力，积极有序推进小火电关闭，实现污染由末端治理向源头预防转变。加大环保执法力度，重点对卫河流域、辉县市水泥群区、卫辉市和凤泉区大气粉尘污染区实行环境综合整治。严防镉、镍等重金属污染。

　　加强就业和社会保障工作。完善和落实小额贷款、税费减免等再就业优惠政策，国有企业下岗职工再就业扶持政策延长三年。力争城镇新增就业6万人，安置下岗失业人员2.3万人，其中“4050”人员6000人。切实加大征缴力度，扩大城镇社会保险覆盖面。推动事业单位养老保险制度改革，进一步完善城市低保制度，做好改制、破产企业医疗保险金的清偿工作。

　　全面发展各项社会事业。以提高公民文化素质和提升城市建筑文化内涵为核心，培育城市文化特色。推进市区中小学布局调整，加快市一中新校区和市外国语中学建设。加大教师资源统筹使用力度，强化薄弱学校师资培训，逐步解决义务教育发展不均衡问题。完善职业教育体系，推进平原职业技术学院、新乡职业技术学院建设，争办全国性劳动技能比赛暨技工技师交流大会。深化医疗卫生体制改革，整顿和规范医疗服务秩序，逐步解决人民群众看病难、看病贵问题。建立和完善多部门通力合作、综合治理人口发展的工作机制，加强出生人口性别比综合治理。认真备战省十运会，深入开展全民健身和体育进社区活动。积极开展双拥工作，努力实现全国双拥模范城“四连冠”。认真做好新闻出版、防震减灾、审计、统计、外事、侨务、档案、史志、气象、测绘、人防等工作。

　　做好安全生产和社会稳定工作。强化企业安全生产责任，健全监督管理体制，实行重大危险源普查制度，减少事故发生。认真贯彻《信访条例》，切实做好信访工作。加强治安防控体系建设，严厉打击刑事犯罪，严密防范境内外敌对势力、邪教组织的渗透、破坏活动，积极争创全省综合治理先进市，建设平安新乡。

　　加强政治文明、精神文明和民主法制建设。坚持党的领导，自觉接受人大及其常委会的法律监督、工作监督和人民政协的民主监督，认真办理人大代表议案、建议和政协委员提案。广泛听取各民主党派、工商联、无党派人士的意见和建议，充分发挥工会、共青团、妇联等人民团体的桥梁纽带作用。以创建全省民族团结进步先进市为重点，做好民族宗教工作。实施公民道德建设工程，强化社会公德、职业道德和家庭美德教育。以征信体系为载体，加快“信用新乡”建设。深入开展普法教育，完善村（居）民自治，强化企业民主管理和厂务公开。加强法律援助工作，依法保障妇女、未成年人和残疾人的合法权益。继续整顿和规范市场经济秩序，实施食品、药品放心工程。

　　在全面完成今年经济社会发展目标任务的同时，着重办好与群众生产生活密切相关的20件实事。

　　一是认真落实好省委、省政府部署的10件实事：1、筹措1亿元，落实好对种粮农民的直接补贴、良种补贴及购买大型农机具补贴；2、筹措9348万元，对农村义务教育阶段贫困家庭学生继续实行免杂费、免书本费和补助寄宿生生活费的“两免一补”政策，切实提高农村中小学公用经费标准，建立农村中小学危房改造长效机制，增强农村义务教育保障能力；3、筹措1.4亿元，完成乡镇卫生院改造任务，全面实行城乡医疗救助制度；4、筹措4.5亿元，改造和建设农村公路，改善农民出行条件；5、筹措2850万元，进一步解决环境污染和农民饮水安全问题；6、筹措2450万元，推进沼气服务站和秸秆综合利用小区建设，提高农村沼气发展水平；7、筹措1159万元，实施农村“村村通”工程和党员远程教育工程，扩大农村广播电视和信息网络覆盖面；8、筹措300万元，对农村劳动力进行职业、技能以及实用技术培训，加快农村富余劳动力转移；9、筹措8385万元，做好城镇就业再就业工作；10、筹措1.2亿元，逐步提高城市居民最低生活保障标准，增加困难群体救助和扶贫开发投入。

　　二是调整财政支出结构，再为我市城乡群众办好10件实事：1、确保市区有劳动能力的“零就业”家庭至少有1人实现稳定就业；2、对城区低保对象家庭学生实行 “两免一补”；3、全部解决市区人均居住面积4平方米以下的特困家庭住房困难；4、市区新建10万平方米经济适用房；5、完成市区58条小街巷和西孟排水沟、王村斜沟、饮马口斜沟、赵定排（和平路至振中路段）治理任务；6、市区新建公厕25座、改造提升120座，建成垃圾中转站10座；7、每个县（市）都要建立一所集职前教育和职后教育于一体的职业教育中心；8、新建农村敬老院10所、改造提升10所，五保户集中供养率达到30%；9、对小型渠道、小桥涵闸、机井等小型农田水利工程建设进行补贴；10、新增通班车行政村150个，行政村通车率达到98%。

四、加强政府自身建设 全力打造人民满意政府

　　建设责任、法治、服务型政府。认真落实《行政许可法》和《全面推进依法行政实施纲要》，严格按法定职责和法定程序行政，保证政府行为合法适当，维护社会的公平与正义。继续深化行政审批制度改革，大力推进政务公开，在公共服务部门全面推行办事公开制度。加强政府门户网站建设，扩大发行《政府公报》。健全重大决策公示、听证制度，推进决策民主化和科学化。认真贯彻《公务员法》，加大公务员教育培训力度，健全绩效考核体系，对不具备基本工作能力和职业操守的公务员进行离岗培训。推进政企分开、政资分开、政事分开、政府与市场中介组织分开，健全适应现阶段经济社会发展需要的宏观管理体制。完善政府责任目标考评制度，增加衡量科学发展、和谐社会建设等方面的指标，切实加强对贯彻落实科学发展观情况的监督检查，加强对解决损害群众切身利益问题的监督检查。严格实行行政首长问责制，落实首问负责制、服务承诺制和限时办结制，加大行政效能监察力度，健全责任、有序、高效的工作推进机制，提高工作效率和服务水平。

　　全心全意为人民谋利益。人民是公务员的衣食父母，我们要时刻抱有感恩之心，真正把群众利益放在高于一切、重于一切、先于一切的位置上，绝不能只看到高楼大厦、歌舞升平，而看不到困难群众的艰难窘迫；绝不能只看到自己的形象、政绩、进退，而看不到基层和群众的所急、所需、所盼。要时刻谨守为民之责，设身处地为群众着想，实实在在解决他们最现实、最关心、最直接的生产生活问题，让最困难的群众看到希望，让每个人都分享到改革发展的成果。对关注民生的各项政策措施实行专项效能监察，确保不折不扣地落实到最需要的群众身上，严禁政策棚架，严禁弄虚作假，严禁以权谋私。

　　切实转变工作作风。始终坚持讲大局、讲原则、讲纪律，任何时候都要以新乡发展的大局为重，以最广大人民群众的利益为重，绝不允许在重大问题上各行其是；任何时候都要牢记自己是人民公仆，站在人民的立场之上，以党的方针政策为指针，坚持原则不动摇，坚决摒弃庸俗作风，绝不允许只代表某个利益集团，为极少数人谋取私利；任何时候都要严格工作纪律，强化责任追究，狠抓工作落实，确保政令畅通，绝不允许推诿扯皮、敷衍塞责。始终追求高标准、高质量、高效率，工作必须雷厉风行，服务必须精益求精，绝不自我原谅，绝不找任何借口。始终增强责任感、使命感、紧迫感，殚精竭虑，发奋图强，努力在区域竞争中把握主动，在扬长补短中争创优势，在超越自我中寻求突破。始终保持蓬勃朝气、昂扬锐气、浩然正气，不争权力大小，不争角色轻重，不争功过是非。始终树立正确的政绩观，不急功近利，不好大喜功，埋头苦干，求真务实，绝不搞造势一时的“政绩工程”。始终牢记“两个务必”，强化廉政建设，反对享受摆阔，杜绝铺张浪费，保持清正廉洁，一心一意为人民谋求更大的福祉。

　　各位代表，展望“十一五”，我们正站在一个新的历史起点之上，面临的任务艰巨而光荣。让我们在以胡锦涛同志为总书记的党中央领导下，高举邓小平理论和“三个代表”重要思想伟大旗帜，坚定信心，团结拼搏，扎实做好各项工作，为在全省率先实现全面建设小康社会的宏伟目标而努力奋斗！
